# A Systematic Review of Emoji: Current Research and Future Perspectives

**DOI:** 10.3389/fpsyg.2019.02221

**Published:** 2019-10-15

**Authors:** Qiyu Bai, Qi Dan, Zhe Mu, Maokun Yang

**Affiliations:** ^1^The School of Journalism and Communication, Renmin University of China, Beijing, China; ^2^Research Center of Journalism and Social Development, Renmin University of China, Beijing, China

**Keywords:** emoji, communication, emotion expression, semantic expression, systematic review

## Abstract

A growing body of research explores emoji, which are visual symbols in computer mediated communication (CMC). In the 20 years since the first set of emoji was released, research on it has been on the increase, albeit in a variety of directions. We reviewed the extant body of research on emoji and noted the development, usage, function, and application of emoji. In this review article, we provide a systematic review of the extant body of work on emoji, reviewing how they have developed, how they are used differently, what functions they have and what research has been conducted on them in different domains. Furthermore, we summarize directions for future research on this topic.

## Introduction

With the widespread application of computing and the development of technology, computer mediated communication (CMC) is infiltrating daily life to a greater and greater extent. It has many advantages, including enhancing the continuity of individual communication (Juhasz and Bradford, 2016), improving the quality of relationships (Pettigrew, 2009; Perry and Werner-Wilson, 2011), and strengthening emotional communication (Derks et al., 2008b). However, the lack of non-verbal cues such as facial expressions, intonation, and gestures in CMC can affect the transmission of information (Archer and Akert, 1977). To address this problem, communicators have devised new non-verbal cues, such as capitalization as a substitute for shouting, multiple exclamation points for excitement, and expression symbols for facial expressions (Harris and Paradice, 2007; Riordan and Kreuz, 2010). These expression symbols make up for the lack of non-verbal cues in CMC (Tossell et al., 2012; Negishi, 2014), and are very well-suited for social media communication (Barbieri et al., 2016c). As a result, emoji, which are a set of expression symbols, came into being.

Emoji are used more and more frequently in network communication, and the way they are used is becoming more and more diversified as well. They not only have unique semantic and emotional features, but are also closely related to marketing, law, health care and many other areas. The research on emoji has become a hot topic in the academic field, and more and more scholars from the fields of computing, communication, marketing, behavioral science and so on are studying them. This paper reviews the developmental history and usage of emoji, details the emotional and linguistic features of emoji, summarizes the results of research on emoji in different fields, and puts forward future research directions.

## Literature Search and Criteria for Inclusion

We started by selecting databases. With reference to the major platforms for related publications, we chose Web of Science and Google Scholar as literature sources. On August 3rd, 2019, we searched Google Scholar and Web of Science using “emoji” as the key word for related literature in English since 1998. Patents, news, book reviews, editorials, letters, and other literature types were excluded and the two databases were combined. After getting rid of the duplicates, we got 167 papers published in 78 journals and and delivered at 33 conferences. We summarized the list of journals and conferences that published articles on emoji more than once, as shown in Table 1. In addition, due to the extensive use of emoji in social networks, we also referred to some online data sources, such as https://emojipedia.org/, https://www.reddit.com/r/EmojiReview/. In this paper, the above contents were summarized and reviewed.

**Table 1:** Statistics for articles on emoji published in journals and delivered at conferences.

**Source**	**Name**	**Amount**
Journal	Computers in Human Behavior	8
	Food Quality and Preference	7
	Food Research International	4
	Discourse, Context & Media	4
	Social Science Computer Review	3
	Plos One	3
	Behavior & Information Technology	3
	Social Media+ Society	2
	Marriage Family Review	2
	Journal of Pragmatics	2
	Frontiers in Psychology	3
	First Monday	2
	Cyber Psychology & Behavior	2
Conference	Association for Computing Machinery	6
	World Wide Web Conference	6
	Association for the Advance of Artificial Intelligence	5
	Workshop on Computational Approaches to Subjectivity, Sentiment and Social Media Analysis	4
	Institute of Electrical and Electronics Engineers	4
	International Conference on Human-Computer Interaction with Mobile Devices and Services Adjunct	3
	International Conference on Knowledge Engineering and Applications	2

## Research Status

In recent years, emoji have become a hot topic for research, with the volume of papers increasing gradually from 2015 and peaking at 2017-2019. Research mainly comes from the fields of computer science and communication science. Marketing, behavioral science, linguistics, psychology, medicine, and education are also involved. Research mostly uses empirical analysis, focusing on the diversity of individuals, cultures and platforms in the use of emoji, the attributes and characteristics of emoji, their functions in communication and the application of emoji in various research directions. Table 2 systematically summarizes the main research fields, research topics, main conclusions and research methods for emoji.

**Table 2:** Main research fields, research topics, main conclusions, research methods, and number of publications for emoji.

**Research fields**	**Research topic**	**Main conclusions**	**Research method**	**Amount (proportion)**
Computer Science	1. Analyzing emotional and semantic meanings of emoji using big data. 2. Switching between emoji and other expression modality. 3. Using emoji for emotional analysis of online data. 4. Using emoji for optimizing computer systems.	1. A series of emotional and semantic lexicons of emoji have been build. 2. A variety of modality transfer and sentiment analysis algorithms have been developed. 3. Some potential applications of emoji, such as enhancing password security, are proposed.	1. Sentiment Lexicon 2. Deep Learning 3. Classification 4. Emotion Recognition 5. System Optimization	51 (30.18%)
Communication	1. The role of emoji in computer mediated communication. 2. The effect of emoji on the user and emoji preference in different contexts.	1. Emoji can make up for the lack of non-verbal clues in CMC, help user convey emotions and meanings and promote online social interaction. 2. Different platforms, cultural backgrounds, linguistic environment and personal characteristics are found to have effects on emoji preferences.	1. Deep Interview 2. Content Analysis 3. Survey	44 (26.04%)
Marketing	1. The impact of Emoji in marketing activities. 2. The impact of emoji on consumers. 3. Whether emoji's advantages in emotional expression can be used to measure users' emotions.	1. The use of Emoji in marketing activities can enhance the appeal of these activities and bring them closer to the younger generation. It can also have an impact on consumers, including optimizing consumer experience, improving purchase intention, and changing perceptions of brands. 2. Emoji can be used to measure users' emotions and depict the portraits of users, and some food-related emoji questionnaires have been developed.	1. Experimental Design 2. Survey	25 (14.79%)
Behavioral Science	1. Descriptive analysis of emoji usage behavior, including users‘ preferences, purpose of emoji and possible factors influencing emoji usage	1. Compared to emoticons, people use emoji more frequently and display a more positive attitude toward them. 2. In general, emoji are used to facilitate communication and interaction and to construct the identification. 3. The use of emoji is influenced by various factors such as platform, individual characteristics and cultural background. Emoji may cause inefficiencies and misunderstandings in practice.	1. Experimental Design 2. Survey 3. Deep Interview	21 (12.43%)
Linguistics	1. The pragmatic functions of Emoji as non-verbal clues. 2. The possibility of emoji functioning as independent languages.	1. In pragmatics, emoji can promote speech acts and interaction. 2. Emoji as a paralinguistic component is equivalent to a morpheme unit. 3. There have been attempts to develop emoji as an independent language, but it they still limited.	Discourse Analysis	10 (5.92%)
Psychology	1. The relationship between user personality traits and emoji use patterns, particularly in the amount of use and specific preferences. 2. Explore the feasibility of using emoji to measure emotions (stress, positivity, negativity, etc.)	1. The use of Emoji is associated with psychological differences (Big Five personality traits, self-monitoring, etc.) 2. Emoji can be used to measure users' emotions and personalities, helping to prevent crises and monitor emotions.	Experimental Design	9 (5.33%)
Medicine	1. Whether the advantages of emoji in interpersonal communication and emotional expression can be applied to the medical field.	1. Emoji can promote doctor-patient communication and improve the health management level of patients. 2. Emoji have also been used to predict mental illness and have shown remarkable accuracy in identifying depression.	Tool Development	5 (2.96%)
Educational	1. The possibility and effectiveness of emoji in education, especially in children's education and online education.	1. The visual features of emoji enable them to overcome language barriers so they can help children learn and understand concepts and help non-English speakers learn English. 2. Emoji used in classroom teaching can help students understand the non-verbal features in CMC communication. 3. Emoji can enhance the effectiveness of communication in online courses.	Experimental Design	4 (2.37%)

In addition, a lot of researches on emoji are cross-field. For example, as emoji are platform-or system-dependent, they are often used in online communication. Due to its visual characteristics or platform differences, there would be emotional or semantic ambiguity in communication. Many researchers from computer science try to solve this problem using a computer method and a series of algorithms or models for semantic disambiguation and sentiment analysis have been developed. Besides, the use of emoji is associated with psychological differences. Some researchers in the field of psychology have also focused on emoji usage to search for the relationship between user's behavior and personality traits. What's more, emoji is used in marketing activities to enhance interaction and promote consumers' willingness to purchase. In order to make better use of this symbol, researchers from the field of marketing draw on relevant theories in the field of linguistics, especially in rhetoric, to enhance the appeal of emoji in marketing activities.

## The Development of Emoji

Emoji originated from smiley, which first evolved into emoticons, followed by emoji and stickers in recent years. Smiley first appeared in the 1960s and is regarded as the first expression symbols. Smiley is a yellow face with two dots for eyes and a wide grin which is printed on buttons, brooches, and t-shirts. By the early 1980s, this symbol had become widespread, emerging as a permanent feature of western popular culture (Stark and Crawford, 2015).

Emoticons were introduced in 1872 and use ordinary punctuation marks from a standard computer keyboard to build up a representation of a face with a particular expression (Zhou et al., 2017). They are a paralinguistic element (Lee and Wagner, 2002; Jibril and Abdullah, 2013) often used at the end of a sentence (Sakai, 2013). Prior to the existence of emoji, users of Instant Messaging (IM) would often use emoticons. Like non-verbal clues in face-to-face communication, emoticons can help clarify intentions in ambiguous contexts (Thompson et al., 2016), express emotions (Walther and D'Addario, 2001; Aldunate and Gonzálezibáñez, 2016; Wall et al., 2016; Esposito et al., 2017) and improve the efficiency of communication (Dunlap et al., 2016). Besides, emoticons possess nonverbal communication functions. They can help those receiving them correctly understand the sender's emotion, attitude, and level of attention (Lo, 2008), bring enjoyment (Chen and Siu, 2017), promote interaction (Aldunate and Gonzálezibáñez, 2016) and community identity (Cho, 2016). In practice, gender, and cultural differences lead to different preferences for emoticon usage (Wolf, 2000; Jack et al., 2009). It has also been suggested that emoticons could be applied to real life, for example in fields such as emotional monitoring (Carvalho et al., 2009; Barbieri et al., 2014), psychological testing (Tan et al., 2018) and designing signs (Sodikin, 2018).

The first set of emoji was released in 1999 and was created by their Japanese originator Shigetaka Kurita (
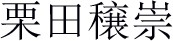
). “Emoji” is a transliteration of the Japanese word 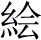 (e=picture) 文 (mo=write) 字 (ji=character)[8]. They are graphic symbols with predefined names and code (Unicode), which can represent not only facial expressions, abstract concepts and emotions/feelings, but also animals, plants, activities, gestures/body parts, and objects (Rodrigues et al., 2017). Possessing similar neural responses to face-to-face communication (Gantiva et al., 2019), using emoji can add extra emotional or contextual meaning to communication, enhance the attractiveness of the message to receivers (Cramer et al., 2016), help users in tone adjustment and conversation management and play a role in managing and maintaining interpersonal relationships (Kelly and Watts, 2015; Chairunnisa and Benedictus, 2017; Albawardi, 2018). On a social level, emoji, as a visual language, make it easier for non-English speaking nations to use English-dominated social media such as Twitter, Instagram and Facebook (Boothe and Wickstrom, 2017). Emoji are widely used in instant messaging, e-mail, social networking and many other forms of CMC (Dresner and Herring, 2010). As indicates, emoji fill the need for non-verbal cues in CMC to express the intentions and emotions behind information (Alshenqeeti, 2016).

In recent years, in order to realize the interpretability of information transmission and better express its meaning, stickers came into being (Zhou et al., 2017). Stickers can help users strategically and dynamically choose the best way to express their emotions, opinions, and intentions and to achieve communicative fluidity (Lim, 2015). At the same time, stickers can be used for strategic motives such as self-presentation, impression management, establishing social existence and maintaining social status (Lee et al., 2016). Besides, responding to a partner with a combination of text and stickers can establish a high level of intimacy (Wang, 2016).

Smiley, emoticons, emoji, and stickers differ in form and content, and have been favored by users in different periods. Smiley, often used in advertisements and product packaging, can encourage positive moods and improve morale (Stark and Crawford, 2015). Unlike emoji, emoticons, and stickers which possess a whole set of characters, smiley is a single symbol rarely used in communication. Emoticons present facial expressions by various combinations of punctuation marks and can be used in CMC. Studies have shown that smiley and emoticons have no difference in information interpretation, but that smiley has a greater impact on individual mood than smiling emoticons (Ganster et al., 2012). Emoji have come to be regarded as an advanced version of emoticons (Aull, 2019), and are superior to emoticons in terms of content richness, input speed and expressiveness (Barbieri et al., 2016b; Rodrigues et al., 2017). Because both act as auxiliary means of communication, emoji and emoticons are completing for similar functions. But the emergence of emoji has been proven to impact the status of emoticons to a certain extent. Compared to emoticons, users use emoji more frequently, with a more positive attitude and a deeper level of identification (Prada et al., 2018). Stickers appeared recently. They are bigger, with static and animated forms, can be added or deleted (emoji rely on Unicode which can't be edited). But stickers can only be sent separately without insertion in text messages (Zhou et al., 2017). Table 3 summarizes the differences between smiley, emoticons, emoji, and stickers.

**Table 3:** The differences between smiley, emoticons, emoji, and stickers.

**Name**	**Time of occurrence**	**Form**	**Content**	**Usage scenarios**	**Unicode**	**Examples**
Smiley	1960s	Static	Single smiley face	Daily life	Without unicode	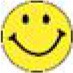
Emoticon	1982	Static	Various facial expressions	Daily life /CMC	Without unicode	^∧^_^∧^
Emoji	1999	Static	Facial expressions, abstract concepts, emotions/feelings, animals, plants, activities, gestures/body parts, and objects	Daily life /CMC	Own unicode	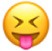
Sticker	After the 21st century	Static/Animated	Texts, facial expressions, abstract concepts, emotions/feelings, animals, plants, activities, gestures/body parts, and objects	Daily life /CMC	Without unicode	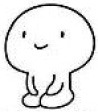

As emoji currently make up the most widely used and standardized symbolic language with the largest number of existing studies, this paper mainly reviews and discusses related research on emoji.

## The Use of Emoji

As non-verbal cues in CMC, emoji are widely used in internet communication. As of March 2019, there were 3,019 emoji in Unicode, with nearly half of all text messages on Instagram containing emoji (Dimson, 2015), and 5 billion of them being used daily on Facebook. In 2015, emoji 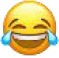 was named the word of the year by the Oxford English Dictionary, indicating emoji's influence in online communication.

### Use Motivation

Simplicity, convenience and conduciveness to emotional expression are the main motivations attracting users to use emoji. Specifically, emoji can help users to express themselves, relax their mood (Kaye et al., 2016) and build their own identity (Ge and ACM, 2019). As contextualization cues (Al Rashdi, 2018), emoji are used in communication to promote interaction (Gibson et al., 2018), including establishing emotional tone, reducing discourse ambiguity, enhancing context appropriateness (Kaye et al., 2016) and intensifying or weakening speech acts (Sampietro, 2019). In addition, emoji are also used to greet (Aull, 2019), and to maintain and enhance social relations while strengthening communication within a platform (Monica Riordan, 2017b). However, some researchers point out that emoji may also be maliciously used for deception (Njenga, 2018).

### Diversity of Emoji Use

In the process of using emoji, the differences between individual characteristics, platforms, cultural backgrounds, and contexts may lead to different understandings. Emoji are also used for some specific topics, such as in sexually suggestive contexts (Thomson et al., 2018). This paper systematically summarizes the differences of emoji in terms of individual diversity, cultural diversity, platform diversity, and their inefficiency in use.

#### Individual Diversity

The use of Emoji is influenced by demographic characteristics and individual psychological characteristics.

First of all, there are significant gender differences. Although males and females understand the function of emoji similarly (Herring and Dainas, 2018), females use emoji more frequently and positively (Prada et al., 2018) while males use more types of emoji (Tossell et al., 2012). However, this trend varies according to communication situation. In public communication, women are more likely to use emoji while in private communication the opposite is true (Chen Z. et al., 2018). In terms of the cognition of emoji, females perceive emoji as more familiar, clear and meaningful (Rodrigues et al., 2017). Male users prefer to use the same emoji to enhance emotional expression (Chen Y. et al., 2018). When men and women use the same emoji, the recipients feel different emotions. Women who send messages containing affectionate emoji are considered more appropriate and attractive than men, and when men send messages containing less affectionate but friendly emoji messages, they are considered more appropriate and more attractive than women (Butterworth et al., 2019).

The use of emoji is also affected by individual psychological differences. This has been shown in research which demonstrates a positive correlation between the frequency of emoji use among Facebook users and their extraversion and self-monitoring traits (Hall and Pennington, 2013), and a negative correlation between positive emoji use and users' emotional distress (Settanni and Marengo, 2015). An emoji-based personality test indicated that the similarity score between emoji and oneself was correlated with emotional stability, extroversion and agreeableness out of the Big-Five personality traits, but not correlated with conscientiousness and openness (Li et al., 2018). Specifically, negative emojis were negatively correlated with emotional stability, while positive emoji were positively correlated with extraversion. In addition, emojis associated with blushing (e.g., 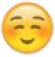) were positively correlated with agreeableness.

As people become more and more enthusiastic about using emoji, some emoji forums emerged, such as https://www.reddit.com/r/EmojiReview/. In the forum, people communicate with each other to explore the various uses and meanings of emoji. With the increase of the need to express individual diversity, people are no longer satisfied with using the existing emoji in the system, but began to create their own expressions and add more personal characteristics to emoji. For example: 
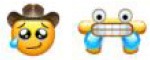
. These are new symbols created by people after recombining existing emoji.

#### Cultural Diversity

Emoji use is structured by a combination of linguistic and social contexts, as well as cultural conventions (Derks et al., 2008a; Park et al., 2013), and is influenced by many factors, such as cultural background, living environment, language environment and user group.

Cultural differences have a significant impact on the use of emoji. Some specific uses of emoji are closely related to cultural background (Park et al., 2014). For example, Finnish, Indian and Pakistani users will use specific emoji according to their own culture (Sadiq et al., 2019). In terms of usage behavior, following Hofstede's cultural dimension model, people in countries with high power distance and indulgence use more emoji representing negative emotions, while people in countries with high uncertainty avoidance, individualism and long-term orientation often use emoji representing positive emotions (Xuan et al., 2016). Specifically, Chinese users are more likely than Spanish users to use non-verbal cues such as emoji and emoticons to express negative emotions (Cheng, 2017). Research has also found that people from Hong Kong and the US use emoji differently on user-generated restaurant reviews websites, which may reflect underlying cultural differences (Chik and Vasquez, 2017). Because of the cultural differences in emoji use, an EmojiGrid was developed for cross-cultural research on food-related emotions, which reliably reflects established cultural characteristics (Kaneko et al., 2019). This difference is not only evident between countries, but also within the same country (Barbieri et al., 2016a).

Emoji use is partly influenced by a range of national developmental indicators (including life expectancy, tax rates, trade and GDP per capita). One line of research using the K-MEANS clustering algorithm found that the most distinctive feature of emoji use in the “first world” (defined here as North America, Western Europe, the Russian Federation, and Australia) was a lack of emotions, while in the “second world” cluster (covering most of South America, Eastern Europe, India, China, Eastern Europe, Morocco, Algeria, and Tunisia) emoji are used in a more specific, emotionally clear way. The “third world” cluster (Angola, Nigeria, Sudan, Jordan, Saudi Arabia, Yemen, Pakistan, Nepal, and the Philippines) uses a balance of positive and negative emoji, and the “fourth world” cluster (made up of certain African countries) uses mostly negative emoji (Ljubešić and Fišer, 2016).

Specific language environments also affect the use of emoji. Emoji show a high degree of context sensitivity in cross-language communication, meaning that they are exceedingly dependent on their linguistic and textual environment (Vandergriff, 2013). For example, research suggests strong similarities in emoji use between Britain and America due to the fact that they both speak English, but there was less similarity when the comparison was made with other languages, such as Italian and Spanish (Barbieri et al., 2016b).

There are also differences in the use of emoji among specific cultural groups. One example of this is a particular style of mobile communication creatively balancing use of emoji, stickers, and text developed by some adults in rural and small towns in Southern China (Zhou et al., 2017). Research has also shown that Japanese teenagers find innovative new ways to use emoji so as to manage their relationships and express themselves aesthetically in a subculturally specific way (Sugiyama, 2015). The use of Emoji is also related to interpersonal relationships (Gaspar et al., 2015, 2016). The more polite and distant the conversation between people, the more abstract, geometric and static the emoji will become. On the contrary, more specific and vivid emoji is used in groups where participants are more sympathetic to a particular topic, more companionate and more intimate (Lin and Chen, 2018).

#### Platform Diversity

Platform diversity is one of the important factors affecting emoji use. The presentation style of emoji on different operating system platforms and the architectural specifications of different network platforms will affect users' preferences for emoji.

Although emoji use Unicode, the presentation style of emoji in IOS, Android, Microsoft and other systems is different due to the influence of different developers (as shown in Table 4). Studies have found that emoji on the IOS platform are more aesthetically attractive, familiar, clear and meaningful than those on the Android platform (Rodrigues et al., 2017). This difference in platform display will lead to misunderstanding and divergence in terms of emoji's emotional and semantic interpretation during cross-platform use (Tigwell and Flatla, 2016). In addition, researchers have studied different network platforms such as Twitter, Facebook and Instagram, and found that users of different platforms have their unique preferences when using emoji. The most popular emoji in one platform may not be popular on other platforms. For example, users tend to use emoji more frequently and positively on Facebook than on Twitter (Tauch and Kanjo, 2016). At the same time, some researchers focus on more marginal community platforms like Gab. In the face of the same event, Gab users tend to publish positive emoji to express irony in text with negative connotations, while Twitter users tend to use emoji to express suspicion (Mahajan and Shaikh, 2019). However, some researchers believe that the use of emoji is generally consistent on all platforms, except for e-mail, which is not suitable for using emoji (Kaye et al., 2016).

**Table 4:** Emoji differences on major platforms.

	**Face with tears of joy**	**Red heart**	**Pleading face**	**Fire**	**Smiling face with heart-eyes**	**Smiling face with smiling eyes**	**Smiling face with hearts**	**Thumbs up**	**Thinking face**
IOS	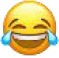	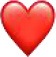	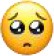	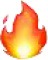	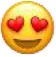	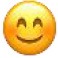	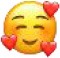	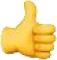	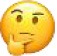
Android	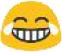	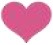	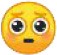	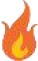	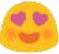	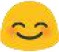	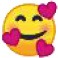	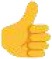	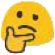
Windows	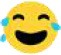	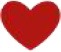	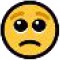	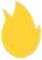	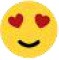	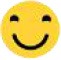	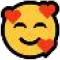	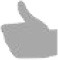	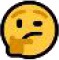

*Source https://emojipedia.org/*.

### Use Inefficiency

Emoji can help users to convey feelings and understand the meaning of a text, but the use of emoji also brings ambiguities in the interpretation of communication, resulting in inefficiency. Although emoji have visual similarity, their interpretation is influenced by cultural background, technical differences and their own visual characteristics (Bich-Carriere, 2019). The specific meanings that users want to express by emoji may be different from their official definitions, resulting in different interpretations of the same emoji (Miller et al., 2016) (Table 5). For example, some people interpret this emoji (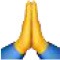) as “prayer” and others interpret it as “clapping hands”. In this case, it is difficult for the two sides to understand each other, which reduces the efficiency of communication. Berengueres and Castro (2017) found that there are differences in understanding negative emoji. For the same negative emoji, the sender's emotional feelings can be 26% different from the receiver's. Research done by Riordan (2017a) shows that the degree of misunderstanding of facial emoji is higher than that of non-facial emoji, but that both are related to the degree of information ambiguity. When used across platforms, the differences in how people interpret emoji emotionally and semantically will increase because of platform display differences (Miller et al., 2016). The difference in how emoji are understood results in inefficiency in communication, leads to the interruption of discourse and destroys interpersonal relationships (Tigwell and Flatla, 2016).

**Table 5:** Common examples of emoji using ambiguity.

**Emoji**	**Name**	**Official definition**	**Misunderstanding**
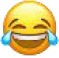	Face with tears of joy	Something is funny or pleasing	Loudly crying face
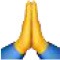	Folded hands	Please or thank you or praying hands	A high five
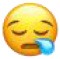	Sleepy face	Tired or sleeping in anime or manga	Crying face
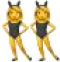	Women with Bunny Ears	An iteration of the Playboy Bunny known in Japan as a Bunny Girl	Friendship, Fun, or “Let's party”
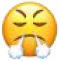	Face with Steam From Nose	Irritation, anger, and contempt	Pride face
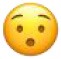	Hushed face	Being hushed by concern or correction	Astonished face
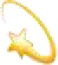	Dizzy	Being dizzy	Fantastic ideas
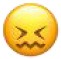	Confounded face	Confused	Frustrated and sad face
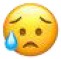	Sad but relieved face	Concern or Anxiety	Crying Face
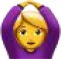	Woman gesturing ok	“OK” sign	Put your hands together as a loving heart
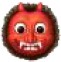	Ogre	Depicts an oni, a kind of hideous ogre in Japanese folklore	Supernatural or figurative beasts and demons
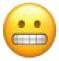	Grimacing face	Nervousness, embarrassment, or awkwardness	Mischievous grimace

## Functions of Emoji

As an important visual symbol in computer-mediated-communication, emoji can express various content, including people, animals, food, activities. Emoji can be used both as an independent language and a non-verbal cue to convey meanings, which is the semantic function of emoji. In addition, emoji also have emotional functions. We have summarized them in Table 6.

**Table 6:** Categories, semantic, and emotional functions of emoji.

	**Category**	**Definition**	**Example**
Content	Smileys and people	Emojis for smileys, people, families, hand gestures, clothing, and accessories.	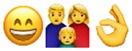
	Animals and nature	Emojis for animals, nature, and weather.	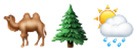
	Food and drink	Emojis for fruit, vegetables, meals, beverages, and utensils.	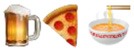
	Activity	Emojis for sports, music, the arts, hobbies, and other activities.	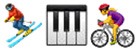
	Travel and places	Emojis for varied scenes, locations, buildings, and modes of transport.	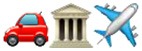
	Objects	Emojis for household items, celebrations, stationery, and miscellaneous objects.	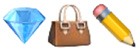
	Symbols	Heart emojis, clocks, arrows, signs, and shapes.	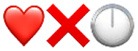
	Flags	Flag emojis, mainly flag emojis of different countries.	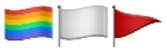
Meaning	Behavioral	Express a behavior, behavioral intentions or activities, such as agree, running, etc.	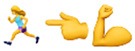
	Non-behavioral	Represent objects, symbols, animals etc.	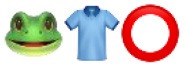
Emotion	Positive	Express positive emotions such as happiness, joy, excitement, etc.	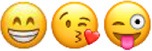
	Neutral	Express moderate emotions. Neither positive nor negative.	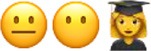
	Negative	Express negative emotions such as sadness, anger, being upset, etc.	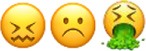

### The Emotional Functions of Emoji

Because they are non-verbal cues with rich emotional meanings, emoji are an important medium for interaction and emotional communication on the Internet.

Emoji can express or enhance emotions (Gülşen, 2016). Jaeger and Ares (2017) analyzed 33 facial emojis and found that most emoji can express one or more emotions. The rich emotional meaning of emoji makes them a key area for researchers who analyze their emotions and develop emoji emotional lexicons. By artificial annotating, Petra et al. (2015) divided emojis into positive, negative and neutral according to their emotional distribution, and found that most emojis were positive, but there were also some emojis which can express irony or satire (Vanin et al., 2013). Due to the subjectivity of human annotating, some researchers have proposed the automatic construction of emoji lexicons. Fernandez-Gavilanes et al. (2018) automatically constructed an emoji lexicon based on the official definitions in emojipedia.

Because of their rich emotional meanings, emoji are often used to express emotions in online communication. In general, users tend to use emoji in positive messages and to use them less in sad or angry messages (Cheng, 2017). Different emoji affect people's attention and responses in divergent ways (Hjartstrom et al., 2019). Although both facial and non-facial emoji can express emotions (Riordan, 2017a), facial emoji outperform non-facial emoji (Jaeger et al., 2019). Using non-facial emoji can bring about positive emotions, especially joy, but it can't change the valence of the message (Riordan, 2017b). Different combinations of emoji also have subtle differences in emotional expression, for example, López and Cap (2017) found that when combining frog emoji or hot beverage emoji with other emojis, there will be subtle but observable emotional changes.

### The Semantic Function of Emoji

In addition to expressing emotions, emoji are also used to convey semantic meanings in communication (Na'aman et al., 2017). They can play the role of non-verbal cues to help understand the overall meaning of messages in CMC (Walther and D'Addario, 2001; Jibril and Abdullah, 2013). There has been a lot of discussion about whether emoji could become an independent language. In addition, due to the diversity and similarity of emoji semantics, many researchers from the field of computing pay attention to the word sense disambiguation task of emoji.

Some research suggests that emoji form an independent language. They have a semantic function and visual rhetoric function, can convey meanings as an independent expressing modality (Jibril and Abdullah, 2013), and, through the combination of different emoji, can express subtler semantics (López and Cap, 2017). Compared with plain text, emoji are richer in semantic meaning (Ai et al., 2017), and have semantic similarity in different languages (Barbieri et al., 2016b). At the application level, Khandekar et al. (2019) developed the social media app called Opico to explore the possibility of “emoji-first” communication, which proved that emoji can be used independently in communication without the need for text. However, some researchers suggest that emoji can't be used as an independent language. Lee et al. (2019) found that emoji are similar to the radicals of Chinese characters. Alshenqeeti (2016) argues that emoji is essentially a form of visual paralanguage. Furthermore, emoji tend to be text-related and rarely used independently. Emoji need to be integrated with the text in order to form a complete meaning (Zhou et al., 2017), helping to enhance the clarity and credibility of the text (Daniel and Camp, 2018). In practice, users tend to use emoji as a supplement to text (Ai et al., 2017; Donato and Paggio, 2017), which also indicates that emoji is a paralanguage.

The meaning of emoji varies according to specific context (Gawne and McCulloch, 2019). Their diversity of semantics and flexibility of interpretation may lead to ambiguity when using them (Jaeger et al., 2019). Therefore, a lot of research focuses on the word sense disambiguation task of emoji. Wijeratne et al. (2017) has developed an Emojinet, which combines emoji and text to eliminate ambiguity. Barbieri et al. (2016c) improved the Skip-Gram model, analyzed the semantics of emoji on twitter, and classified them based on semantic similarity.

## Research Fields Regarding Emoji

Emoji have both emotional and semantic functions and are popular in computer-mediated-communication. Researchers from different fields have studied emoji from different perspectives, including computer science, communication, marketing, behavioral science, linguistics, psychology, medicine, and education.

### Computer Science

Research in the field has focused on using emoji for emotional analysis of UGC data, the conversion of emoji to other expression modality, and using emoji for optimizing computer systems.

#### Sentiment Analysis

With the significant growth of UGC data on the Internet, sentiment analysis which aims at changing this data into valuable asset for decision making, has become increasingly important (Al-Azani et al., 2018). As emoji are widely used in expressing emotions, they have become an effective means of sentiment analysis (Hogenboom et al., 2013; Cappallo et al., 2015). A number of studies have confirmed the effective performance of emoji in sentiment analysis (Sari et al., 2014; Cahyaningtyas et al., 2017; Felbo et al., 2017; LeCompte and Chen, 2017). Besides, emoji-based sentiment analysis is language-independent and exhibits cross-language validity (Guthier et al., 2017), for example, Al-Azani et al. (2018) found that emoji can also be used in analyzing the sentiment of Arabic tweets. However, other studies have shown that using emoji in sentiment analysis leads to higher emotional scores, and that this effect is more pronounced in positive comments (Ayvaz and Shiha, 2017).

Many studies have provided algorithms and models for emoji-based sentiment analysis, which mainly uses two kinds of techniques, sentiment lexicon, and machine learning. The sentiment lexicon approach focuses on building an emoji emotional lexicon to support text sentiment analysis. By human annotating, Petra et al. (2015) has classified 751 commonly used emoji and built an emoji lexicon based on the positivity of emoji. But because there are so many emoji, some researchers have come up with ways to build emoji dictionaries automatically. Jiang et al. (2015) proposed an emoticon space model to automatically match emotional tags for emoji. Kimura and Katsurai (2017) assigned multi-dimensional emotional vectors to emoji by calculating the co-occurrence frequency of emoji and emotional words in WordNet-Affect. Aoki and Uchida (2011) have also automatically generated emoji vectors based on the relationship between emotional words and emoji. By using the Word2Vec clustering method, Mayank et al. (2016) divided emoji into clusters which represent different human emotions. The machine learning method refers to train sentiment classifiers based on a corpus in order to analyze the sentiments of text (Wang et al., 2012). Machine learning can be divided into supervised learning and unsupervised learning. They are different in that the former needs a human annotated corpus while the latter doesn't. The effectiveness of using emoji as a way of training classifiers has been proven (Hallsmar and Palm, 2016) and furthermore it has been shown that emoji outperform emoticons (Redmond et al., 2017). An example of supervised learning is the emoticon smoothed language model (ESLAM) proposed by Liu et al. (2012), which classifies twitter based on a model trained by a human annotated corpus. A lot of research has focused on unsupervised learning (Li et al., 2018), and constructed sentiment analysis models trained automatically using emoji data sets. Chen Y. et al. (2018) trained sentiment classifiers by via bi-sense emoji embedding and attention-based long short-term memory network (LSTM) in order to analyze the sentiment of messages on Twitter. Wang et al. (2016) designed a hybrid sentimental entity recognition model (HSERM), which classifies emoji into four different emotional categories, and then categorizes the emotional data based on the model. Some research has focused on the ironic features of emoji and developed an irony detection model for emoji in order to improve the accuracy of sentiment analysis of tweets (Reyes et al., 2013; Prasad et al., 2017; Singh et al., 2019).

#### Modality Transitions

The visual features and Unicode basis of emoji make them an independent expressive modality that is different from text and pictures (Cappallo et al., 2019). A lot of research focuses on conversion between emoji and other modalities such as text, picture and video.

For example, Emoji2Video offers a way to search for videos using emoji (Cappallo et al., 2015). Later research has focused on the shift from other modalities to emoji. Because of the correlation between emotional categories in text and users' emoji selections, Hayati and Muis (2019) and Zanzotto and Santilli (2018) proposed two different ways to predict emoji based on text. Kim et al. (2019) developed Reeboc, which can analyze chat content, extract different emotions or topics, and then, based on this, recommend emoji to users. The practice of text-based emoji prediction has also been validated in other languages, such as Hebrew (Liebeskind et al., 2019).

#### System Optimization

Emoji have played a role in improving the performance of computer hardware and software. For example, emoji can be used to achieve diverse in-car interaction design. In order to optimize the functions of the central rear-view mirror, researchers suggest that passengers emotions can be fed back to the driver through emoji and other elements, which can enhance mutual understanding between driver and back-seat passenger (Chao et al., 2019).

Furthermore, emoji can also be applied in the area of password security. Kraus et al. (2017) came up with the EmojiAuth project, exploring how the use of emoji affects the availability of mobile authentication and user experience by adding emoji into passwords. Compared with the Standard PIN (Personal Identification Number) input, a password containing emoji is easier to remember and, thus, emoji-based authentication is a practical alternative to traditional PIN authentication.

### Communication

In the field of communication, research on emoji mainly focuses on two aspects: one is emoji's emotional and linguistic functions in CMC, the other is how different factors, such as individual characteristics, cultural background and system platform, influence users' preferences for emoji use.

Emoji make up for the lack of non-verbal cues in CMC, and play an auxiliary role in conveying emotion (Gülşen, 2016), expressing semantics (Walther and D'Addario, 2001), and promoting interpersonal communication (Gibson et al., 2018). For example, Jaeger and Ares (2017) analyzed the emotional attributes expressed by 33 facial emojis, and found that most emoji contained one or more emotional meanings. Based on their emotional distribution, Petra et al. (2015) classified emojis into positive, neutral and negative and found that most emojis express positive emotions. Similar studies have found that users tend to use more emoji more in positive messages than negative messages. Both facial and non-facial emoji exhibit a great deal of ability when it comes to expressing emotions (Herring and Dainas, 2018; Jaeger et al., 2019). At the same time, different combinations of emojis can enrich the meanings of emotional expression. López and Cap (2017) studied how emotions change when different combinations of emojis were used. More research in this area is referred to in section The Emotional Functions of Emoji of this paper.

Individual use of emoji is influenced by many factors. The existing research can be divided into three categories: individual characteristics, cultural background and system platform. First, the use of emoji is strongly influenced by demographic characteristics such as gender and age of users. Women use them more frequently and men use them more abundantly (Tossell et al., 2012; Prada et al., 2018). Women use emoji more in public communication, but less in private communication (Li et al., 2018). In terms of social cognition, emoji with stronger emotional meanings are considered more appropriate and lovely for women than for men, while emoji with weaker emotional meanings but friendlier meanings are considered more appropriate for men (Derks et al., 2008a). Secondly, the use of emoji is closely related to the user's cultural background. Users in different countries will use emoji with specific national or ethnic meanings (Gaspar et al., 2016). Finnish users introduce “sauna,” Hindus use “Happy Diwali” and Pakistanis use Namaz symbols for emoji design. Users in different countries tend to use emoji differently. Chinese people use emoji and emoticons more often than Spaniards (Lin and Chen, 2018). Emoji show a high degree of contextual sensitivity and different language types influence the use of emoji. For example, the use of emoji displays a strong correlation among English-speaking countries, while displaying lower correlation among other languages (such as Italian and Spanish) (Barbieri et al., 2016b). Finally, different system platforms also lead to differences in emoji usage. Although emoji has a Unicode in the operating system platform, users show emoji differently in IOS, Android and Microsoft operating systems due to the limitation of these software's developmental compatibility (Cramer et al., 2016). Different social networking platforms such as Twitter, Facebook, Gab and Instagram also have their own particular patterns of emoji usage. For example, users tend to use emoji more frequently and positively on Twitter than on Facebook (Hall and Pennington, 2013). Gab users tend to use positive emoji to express negative emotions, thus showing irony (Settanni and Marengo, 2015). More research is referred to in section Diversity of Emoji Use of this paper.

### Marketing

Due to their visual and emotional attributes, emoji can be used in marketing activities. Emoji play an important role in attracting attention, stimulating social interactions and enhancing the experience of consumers, along with their willingness to purchase (Das et al., 2019). So it is hardly surprising that emoji are frequently used in consumer interactions (Lee et al., 2014; Negishi, 2014). Furthermore emoji are also used to depict consumer emotions (Li et al., 2014). Their dominance in emotional expression makes them an effective tool to measure user's emotions.

Textual paralanguages like emoticons and emoji, can influence the cognition and behavior of consumers in marketing activities (Luangrath et al., 2017; Manganari and Dimara, 2017; Urumutta Hewage et al., 2018), for example, the presence of emoji on food packaging can influence children's dietary choices (Siegel et al., 2015; Luangrath et al., 2017). It has been found that using emoji can enhance the explanatory power, attractiveness, creativity and innovation of marketing activity. With the introduction of emoji in online marketing, more young people are attracted (Yakin and Eru, 2015). Ge and Gretzel (2018) indicate that social media influencers (people who take on the dual roles of marketer and active user of social media) can initiate online interaction by presenting emoji individually or in combination, which can attract consumers to participate in interactions.

Emoji can also be a way of reflecting consumers' emotions, describing user's profiles (Moreno-Sandoval et al., 2018), and especially monitoring the emotions users feel toward products, brands, and services (Rathan et al., 2017, 2018; Phand et al., 2018; Moussa, 2019). It has been found that gender, age and frequency of usage do not affect consumers' ability to describe and distinguish stimuli with emoji (Jaeger et al., 2018b), and certain emojis can help consumers better differentiate product samples (Schouteten et al., 2019). In addition, emoji and emoticons are considered simple and intuitive ways to express food-related emotions (Vidal et al., 2016). Marketers use emoji questionnaires as a common tool to measure user's emotions (Jaeger et al., 2017b, 2019), especially children's food preferences and emotional responses (Gallo et al., 2017; Swaney-Stueve et al., 2018; Lima et al., 2019).

However, some researchers point out that although emoji show more discriminability and simplicity than emotional words in emotional measurement, their multiple meanings could pose a barrier to the survey. Therefore, emoji questionnaires can't directly replace the existing text-based forms of sentiment survey directly. They can, however, act as a complement to the current form (Jaeger et al., 2017a, 2018a).

### Behavioral Science

In the field of behavioral science, research on emoji focuses on three aspects: motivation, preference and influencing factors. There has been abundant research focusing on the motivations behind emoji usage. This research has found that emoji are used for managing and maintaining interpersonal relationships (Chairunnisa and Benedictus, 2017; Riordan, 2017b; Albawardi, 2018), expressing oneself (Kaye et al., 2016), constructing personal identity (Ge and ACM, 2019), facilitating communication and enhancing interaction (Gibson et al., 2018) in interpersonal communication. As a contextual cue, emoji can help users establish an emotional tone, reduce the ambiguity of semantic expression and improve appropriateness relative to context (Kaye et al., 2016). There are two aspects of emoji usage preference. One is users' selection of emoji content and the other is the degree to which there is a match between emotions expressed by emoji and real sentiments. For example, users in different countries introduce elements which are representative of their countries into emoji (Sadiq et al., 2019); and users of one social networking platform prefer to use positive emoji in negative texts to express irony (Kaye et al., 2016).

### Linguistics

In the field of linguistics, research focuses on the pragmatic functions of emoji and the possibility that they could become an independent language. Emoji have been identified as having semantic properties, and can be used both as an independent language and as a component of a paralanguage providing users with a means of communication and promoting speech acts and interaction (Jibril and Abdullah, 2013; Alshenqeeti, 2016; Na'aman et al., 2017). There are pros and cons regarding whether emoji can become an independent language. Some researchers believe that emoji possess visual rhetoric and text functions and have more subtle semantics and for this reason deem that emoji can independently express meaning (Jibril and Abdullah, 2013; López and Cap, 2017). An application was developed to verify the possibility of emoji-first communication (Khandekar et al., 2019). Other researchers think emoji can't be regarded as an independent language because their meaning largely depends on surrounding text, and only when they are combined with the text can complete semantics be expressed (Zhou et al., 2017).

### Psychology

Studies in this field mainly focus on two aspects. One is the relationship between individual psychological characteristics and emoji usage, and the other is the introduction of emoji into the scale design and the implementation of new psychological measurement tools. Emoji usage was found to be closely related to some psychological traits such as the big five personality traits, self-monitoring, emotional stress, and others (Derks et al., 2008a; Hall and Pennington, 2013; Li et al., 2018). For example, research has shown that frequency of emoji use correlates with emotional stability, extroversion and agreeableness in the big five personality traits, but not with conscientiousness and openness (Li et al., 2018). At the same time, some studies have attempted to introduce emoji into psychometric scales and have achieved good results in actual measurements (Marengo et al., 2017; Phan et al., 2019).

### Medicine

In the field of medicine and public health, studies on emoji mainly focus on correcting personal behavior and improving doctor-patient communication. Emoji can be used to guide people's behavior regarding health, and it has been shown that using emoji can reinforce correct behavior when it comes to hand hygiene monitoring (Gaube et al., 2018). Furthermore, using emoji can improve communication between doctors and patients and also enhance patients' abilities to manage their own health (Balas et al., 2012; Troiano and Nante, 2018). Some researchers suggest developing a set of emoji specifically to be used for patient care, which could help patients better understand and communicate the challenges they face in health management (Skiba, 2016). In addition, emoji can be used for the identification and prediction of mental illness due to their strength in emotional expression. Marengo et al. (2019) has introduced emoji into the process of depression assessment and found that accuracy of identifying depression was significantly improved.

### Education

In the field of education, research focuses on the impact of emoji on learning efficiency. It has been found that the use of emoji in classroom activities will help students better understand what they have learned (Brody and Caldwell, 2019), especially in computer-mediated teaching (online learning Dunlap et al., 2016). Emoji can help young children understand abstract concepts such as security, interpersonal management and emotions and also improve their ability to express themselves (Fane, 2017; Fane et al., 2018).

## Future Directions for Research

### The Relationship Between Emoji and Real Sentiments

Understanding users' real emotions when they use emoji is important for future research. At present, it is difficult to accurately measure participants' true reactions through self-reporting. Categorizing emotions by amassing a corpus using big data is unable to depict users' complex emotions such as are expressed by emoji at a more detailed level, for example emotions such as shame, anger and so on. Therefore, we hold the opinion that in the future, researchers can use some psychological methods in the corpus test to measure the physiological indexes of participants with professional equipment such as nuclear magnetic resonance, electroencephalography and multipurpose polygraphs to depict users' real emotions more accurately. Future research could also benefit from a more qualitative approach, such as interviews and case studies to learn about emoji use in the context of real-world communication. In practice, some researchers suggest that video and screen shots can be used in concrete operations to observe and record users' choices of emoji during communication (Gibson et al., 2018). We believe that observing whether users' actual facial expressions differ from their selected emoji emotionally in communication can help researchers understand users' psychological mechanism in communication.

### Factors Influencing User Preferences in Emoji Use

At present, research focuses on the description of users' preference for emoji, but fails to go deeply into the underlying reasons. Emoji such as “heart” (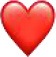) and “tears of joy” (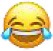) were found to be more popular, but whether their popularity is related to specific cultural traits has not been studied. Users' preferences for emoji are influenced by many factors such as contextual information, interpersonal relationships, familiarity with emoji and personal interpretations other than official definitions, which are all worthwhile factors to explore.

### Sticker's Impact on Emoji

The emergence and widespread use of stickers has impacted the status of emoji, and some research has begun to improve the user experience of stickers (Shi et al., 2019). Whether stickers will replace emoji is an interesting topic for researchers. Under the impact of stickers, how to further enhance emoji's performance in emotion and semantic expression and improve user experience is also a direction worth exploring.

### The Relationship Between Emoji Usage and Social Development

As part of popular culture, the development and use of emoji reflects specific political and cultural characteristics. Many researchers have interpreted emoji's social influence from different perspectives. For example, some uncivilized use of emoji can harm public consciousness, a point which is not yet appreciated by the public (Zerkina et al., 2017). Other researchers believe that the popularity of emoji reflects multicultural communication and cultural globalization (Skiba, 2016), and that there is some unconscious power behind the use of non-verbal cues like emoji (Elder, 2018), which strengthen the inequality and exploitation of our social system (Stark and Crawford, 2015). For example, Leslie (2019) argues that the quantitative use of emoji in the workplace (such as the use of emoji to give ratings) has turned the employee into something like an on-the-shelf item in a digital economy warehouse, affecting their freedom.

The democratization of emoji selection and Unicode should also be discussed. Emoji of different skin colors have been introduced to address the lack of racial representation (Sweeney and Whaley, 2019). In addition, the Unicode consortium recently approved emoji that specifically refer to menstruation, which is seen as a step toward getting rid of “menstrual shame,” reflecting that women's rights are on the increase. Therefore, future research can explore the deeper meaning of emoji use from different perspectives, especially the links between emoji use and political movements, subcultural groups, and social inequality.

## Summary

This paper systematically reviews related research on emoji, aiming to provide a global perspective and clues for researchers interested in emoji. This paper summarizes the developmental process, usage features, functional attributes, and fields of research related to emoji. Emoji developed from emoticons, and have both emotional and semantic functions. The use of emoji is influenced by and varies according to factors such as individual circumstances, culture, and platforms. Ambiguity and misunderstanding may occur in different situations and cultural backgrounds. From the perspective of many fields (communication, computing, behavioral science, marketing, and education), this paper comprehensively combs the research topics, methods and tools used in studies related to emoji, systematically summarizes the research status of emoji in various fields, and puts forward some new perspectives for future emoji research such as emotional association, use preference, new modalities and impacts on society.

## Data Availability Statement

The datasets generated for this study are available on request to the corresponding author.

## Author Contributions

QB contributed conception and design of the study. QB, QD, ZM, and MY wrote sections of the manuscript. All authors contributed to manuscript revision, read, and approved the submitted version.

### Conflict of Interest

The authors declare that the research was conducted in the absence of any commercial or financial relationships that could be construed as a potential conflict of interest.
